# Functional neuroimaging of sensorimotor cortices in postmenopausal women with type II diabetes

**DOI:** 10.1117/1.NPh.7.3.035007

**Published:** 2020-09-02

**Authors:** Stacey L. Gorniak, Victoria E. Wagner, Kelly Vaughn, Jonathan Perry, Lauren Gulley Cox, Arturo E. Hernandez, Luca Pollonini

**Affiliations:** a University of Houston, Department of Health and Human Performance, Houston, Texas, United States; b University of Houston, Department of Psychology, Houston, Texas, United States; c University of Houston, Department of Engineering Technology, Houston, Texas, United States

**Keywords:** functional near-infrared spectroscopy, aging, cortical oxygenation, motor dysfunction, tactile sensation

## Abstract

**Significance**: Deficits in sensorimotor function in persons with type II diabetes mellitus (PwDM) have traditionally been considered a result of peripheral nerve damage. Emerging evidence has suggested that factors outside of nerve damage due to type II diabetes mellitus, such as impaired hemodynamic function, contribute significantly to both sensory and motor deficits in PwDM.

**Aim**: The focus of the current study was to evaluate functional cortical hemodynamic activity during sensory and motor tasks in PwDM.

**Approach**: Functional near-infrared spectroscopy was used to monitor oxyhemoglobin (HbO) and deoxyhemoglobin (HbR) across the cortex during sensory and motor tasks involving the hands.

**Results**: Decline in HbO across sensory and motor regions of interest was found in PwDM with simultaneous deficits in manual motor tasks, providing the first evidence of functional cortical hemodynamic activity deficits relating to motor dysfunction in PwDM. Similar deficits were neither specifically noted in HbR nor during evaluation of sensory function. Health state indices, such as $A_{1c}$, blood pressure, body mass index, and cholesterol, were found to clarify group effects.

**Conclusions**: Further work is needed to clarify potential sex-based differences in PwDM during motor tasks as well as the root of reduced cortical HbO indices but unchanged HbR indices in PwDM.

## 1 Introduction

Nearly 24% of the 40 million individuals in the United States over the age of 60 are currently living with type II diabetes mellitus (DM).^1^ Persons with DM (PwDM) experience decline in hand/finger sensorimotor function as compared to healthy individuals;^2^^–^^4^ however, self-awareness of these changes is low.^5^ Reduced functional hand use has been associated with a loss of independent living and reduced quality of life in PwDM.^6^^,^^7^ Tactile dysfunction due to peripheral neuropathy (PN) has been implicated as the primary cause of motor deficits in PwDM;^8^^–^^11^ however, our recent work has demonstrated that motor changes in PwDM occur independent of tactile impairment, unrelated to disease duration and severity.^12^ Our data point toward other factors, such as alterations in motor unit structure–function and reduced hemodynamic function of muscle and the cortex as underlying mechanisms for these sensorimotor changes.^13^ Due to the confluence of multiple systemic changes in the body in PwDM, the contribution of impaired or altered cortical function to sensorimotor dysfunction prior to PN diagnosis in PwDM is fully plausible.

Altered hemodynamic responses due to both micro- and macrovascular changes have been implicated as a potential source of global motor changes in PwDM.^14^ Specifically, endothelial dysfunction within dermal and muscle tissues in adult PwDM has been related to altered motor behaviors, including abnormal force production.^13^^,^^15^^,^^16^ Currently, it is unclear whether hemodynamic function of cortical tissues, as evidenced by abnormal cortical activation patterns, also occurs in PwDM. Correspondingly, our hypothesis is that altered hemodynamic function of the cortex occurs during tactile sensory and manual motor tasks. Further, these altered hemodynamic patterns may directly relate to poor tactile detection thresholds and motor performance in PwDM. In particular, postmenopausal women may experience significant deterioration of both hemodynamic function and resultant sensorimotor behaviors given the disproportionate risk of cardiovascular complications in women with DM as compared to men with DM and persons without DM.^17^^,^^18^ Accordingly, the focus of this study was to evaluate changes in cortical oxygenation indices of postmenopausal women both with and without DM during (1) tactile stimulation of the hands and (2) during manual isometric submaximal force production tasks.

We expected to see between-group changes in cortical oxygenation indices, such as oxygenated hemoglobin (HbO) and deoxygenated or reduced hemoglobin (HbR), during tactile stimulation of the hands and during isometric submaximal force production tasks (Hypothesis #1). Additionally, based on our previous work, we expected to see between-group differences in tactile sensitivity and in the temporal structure of force variability (Hypothesis #2). No hypotheses regarding changes in cortical hemodynamic function with disease state were developed *a priori*, as investigation of cortical hemodynamic function with disease state was an exploratory aim of this study. To examine these hypotheses, we measured cortical hemodynamic activity with functional near-infrared spectroscopy (fNIRS) alongside individual task performance with the goals of (1) detecting differences between PwDM and controls and (2) evaluating the relationship between cortical hemodynamic activity and task performance.

## 2 Materials and Methods

### 2.1 Participants

Twenty-one self-declared right-handed postmenopausal women with DM and 21 self-declared right-handed age- and sex-matched healthy controls volunteered to participate in the study, see Table 1 for demographics. Handedness was confirmed by the Edinburgh Inventory,^19^ ranging from a laterality quotient (LQ) of –100 (strong left-handedness) to +100 (strong right-handedness). Participants had an LQ average of +88 and had no previous history of trauma to the upper limbs. Study participants were excluded if they reported a history of neurological and/or musculoskeletal disorders (Parkinson disease, Huntington’s disease, polio, multiple sclerosis, stroke, traumatic brain injury, carpal tunnel syndrome, rheumatoid arthritis, monoclonal gammopathy of undetermined significance, paraproteinemic demyelinating neuropathy, myasthenia gravis), a history of amputation, a history of major surgical intervention to the upper extremity, or hereditary or compression neuropathies. In accordance with the Declaration of Helsinki, participants provided informed consent according to the regulations established by the Institutional Review Board at the University of Houston (protocol #15615-01). Data collection processes failed on five participants (e.g., a reliable fNIRS signal was not detected). These were the following participants: controls 2, 7, 10, and 21 as well as PwDM 19. This resulted in a sample size of 17 controls and 20 PwDM. Data from those participants have been excluded from these analyses. *Post-hoc* power analyses using G*Power 3.1.94 indicated that the primary effect size found (d=0.951) in HbO data with the sample sizes of 17 controls and 20 PwDM provided for a *post-hoc* power of 0.88, well above the 0.8 power threshold for traditional power analyses.

**Table 1:** Demographic and clinical characteristics of DM participants.

Participant #	Age (years)	Menopausal age (years)	BMI ($\mathrm{kg}/m^{2}$)	DM duration (months)	$A_{1c}$ (%)	Total cholesterol (mg/dL)	Systole (mmHg)	Diastole (mmHg)
1	63	50	27.4	60	6.7	—	151	81
2	79	45	28.3	144	7.9	—	155	75
3^a^	65	50	40.7	120	7.1	—	145	97
4^b^	66	50	29.3	186	8.7	199	111	62
5^a^	64	40	44.1	60	6.2	109	180	91
6	60	50	37.5	387	10.4	224	161	78
7^a^	60	55	33.7	245	8	143	130	70
8	57	49	36.9	41	8.6	176	130	88
9	73	60	25.3	201	6.8	125	167	78
10	68	23	31.8	168	5.7	219	164	89
11	70	45	26.9	200	6.1	266	130	71
12	62	38	32.4	36	6.2	189	124	70
13	67	45	30.2	1	8	144	158	97
14^a^,^b^	66	45	31.4	262	6.3	175	142	75
15^a^	69	55	42.3	298	8.4	185	139	63
16	58	51	32.8	95	7.4	143	153	89
17^a^	55	27	38.6	385	7.4	126	133	68
18	67	25	30.5	1	7.7	183	148	73
19^a^	71	52	42.9	196	8.5	173	105	60
20	69	27	36.3	149	8.7	187	202	100
21	60	37	30.1	1	6.7	183	179	111
Mean	65	43	33.8	154	7.5	175	148	80
SD	6	11	5.6	117	1.2	39	23	14
Controls	67±6	50±7	24.1±4.5	N/A	5.3±0.3	200±43	147±21	86±14

Note: SD, standard deviation; —, data collection failure.

a Indicates a clinical diagnosis of diabetic peripheral neuropathy.

b Indicates a history of Prempro Rx (in addition to three control participants).

### 2.2 Health Status Data

Blood pressure, cholesterol, and glycated hemoglobin ($A_{1c}$) values were assessed for all study participants onsite. Cholesterol and $A_{1c}$ values were assessed using a commercially available point of care evaluation kit (Cardiocheck+ and $A_{1c}$ Now+ kits, PTS Diagnostics, Indianapolis, Indiana). Blood pressure was measured using a commercially available device (Omron Intellisense 10 series Blood Pressure Monitor, Model BP785, Bannockburn, Illinois). The presence of PN (PN status) was determined by abnormalities on either clinical examination or electromyography/nerve conduction velocity (EMG/NCV) testing (per physician). A brief menopause questionnaire was also administered regarding several aspects of menopausal characteristics (e.g., age at onset of menopause, hormone replacement therapy history, etc.). All study participants declared themselves to be postmenopausal with 11 participants claiming a history of hormone replacement therapy (five with a history of Prempro use).

### 2.3 Tactile Sensory Evaluation

The Semmes-Weinstein monofilament test was used to evaluate tactile sensation of the dominant hand.^20^ Monofilament testing sites included the tip of the thumb/digit 1 (median nerve), the tip of digit 5/hypothenar eminence (ulnar nerve), and dorsal aspect of the thumb (radial nerve). During the test, participants kept their eyes closed and verbally indicated if and where they perceived monofilament touch. The monofilament size was increased until the subject was able to detect its touch a minimum of two times at the same location.

### 2.4 Maximal Force Production

Participants were asked to perform a series of force production tasks consisting of precision pinching (use of digits 1 and 2) at maximal strength using the dominant (right) hand. Maximal pinch evaluation was conducted using a Biometrics Pinch Dynamometer and wireless DataLOG system (Precision Pinchmeter model P200, DataLOG model MWX8, Biometrics Ltd, Newport, UK). Three trials were collected with the dominant hand, and average maximal force production values (MVC) were calculated. During testing, the P200 device was placed 16 in. anterior to the patient torso, 6 in. away from the midline of the body toward the dominant hand. The wrist orientation was such that the hand was in a neutral position during testing.^4^^,^^5^^,^^21^ During testing, the P200 device was held vertically with the nondominant hand.

### 2.5 Experimental Tasks

**Fig. 1:**
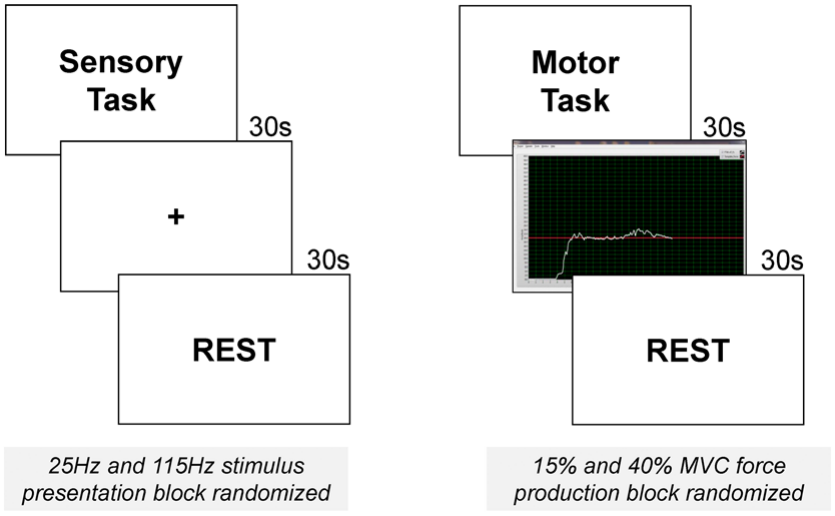
Illustration of experimental stimuli during the vibrotactile stimulation and motor performance tasks during fNIRS testing. Subjects viewed a fixation cross during vibrotactile stimulation blocks; they viewed real-time feedback on their force production during motor blocks. The order of stimulus presentation was block randomized within stimulation type. Sensory stimulation tasks always occurred prior to motor tasks.

Each subject was asked to perform a series of sensory simulation and manual motor tasks executed in a specific order (all sensory tasks prior to all motor tasks) and interleaved by 30-s periods of rest, see Fig. 1 for details. Presentation of visual stimuli, timing, and task-specific synchronization TTL signals was controlled via E-prime 2.0 (Psychology Software Tools, Inc., Sharpsburg, Pennsylvania). During sensory stimulation tasks, digits 1 and 2 of the dominant (right) hand were stimulated with a tendon vibrator device (VB115 Techno Concept Tendon Vibrator Kits, Techno Concept, France) at an amplitude of 1 mm at two frequencies: 25 and 115 Hz. Stimulation frequency presentation was block randomized. Three stimulation trials (lasting 30 s each with 30 s washout periods in between) were presented per stimulation frequency.

During the manual motor task, participants used a precision pinch grip to exert an isometric force against a set of force transducers, see below for details. Participants were instructed to match their pinch force to the target force line as accurately as possible. Two different force levels were tested for the dominant (right) hand (15% MVC and 40% MVC), see Sec. 2.5.1 for details. Three trials of 30 s each were performed with 30 s of rest/washout periods between each block. Force level order (15% or 40% MVC) was block randomized.

#### 2.5.1 Submaximal force production task

Briefly, the task involved using digits 1 and 2 in a precision pinch grip to produce a constant level of pinch force with feedback from a computer screen. All forces and moments of force produced were recorded simultaneously using two identical six-component force–moment transducers (Nano-25 transducers; ATI Industrial Automation, Garner, North Carolina). Instrument details have been published.^4^^,^^5^^,^^21^ No contact of either transducer was permitted prior to trial onset. Two different force production levels were tested: 15% and 40% MVC. MVC values for pinch forces were determined from maximal dynamometry testing (see above). Each participant performed three trials for each condition, lasting 30 s each. Force production order was block randomized. Subjects were not asked to bear the weight of the object, instead all kinetic testing devices were suspended so that participants could easily touch and exert force on the devices without movement of the hand/fingers/forearm (SnakeClamp, Riner, Virginia).

Transducer signals were amplified and multiplexed using ATI hardware prior to being routed to an analog-to-digital converter (via cDAQ-9174 chassis and NI-9205 input modules, National Instruments, Austin, Texas). A customized Labview program (National Instruments) was used for data acquisition and customized MATLAB^®^ (Mathworks Inc., Natick, Massachusetts) programs were written for data processing. Signals were sampled at 100 Hz and low-pass filtered at 10 Hz using a second-order, zero-lag Butterworth filter.^4^^,^^5^^,^^21^ The force data of interest consisted of the final 25 s of each trial.^4^^,^^5^^,^^21^ Participants were required to reach and maintain the indicated force production level within the first 5 s of each trial. Force data of the three trials per condition were averaged for kinetic analyses.

#### 2.5.2 Kinetic analyses

Submaximal data were analyzed with respect to both linear and nonlinear measures. Linear measures of performance included root mean square error (RMSE) of the force output relative to the target and the coefficient of variation (CV). The structure of force output variability was quantified via approximate entropy (ApEn) and detrended fluctuation analysis (DFA). Center of pressure (COP) data was determined via analysis of the measured force and torque data, as described by Zatsiorsky.^22^ The mean area of the COP was calculated by using an 85% best fit ellipse to the COP data using principal component analysis.^23^ The mean area of this ellipse was used as a measure of consistent digit placement and fingertip roll throughout the duration of skin to object contact in manual tasks.^24^

### 2.6 Cortical Hemodynamics Measurements

**Fig. 2:**
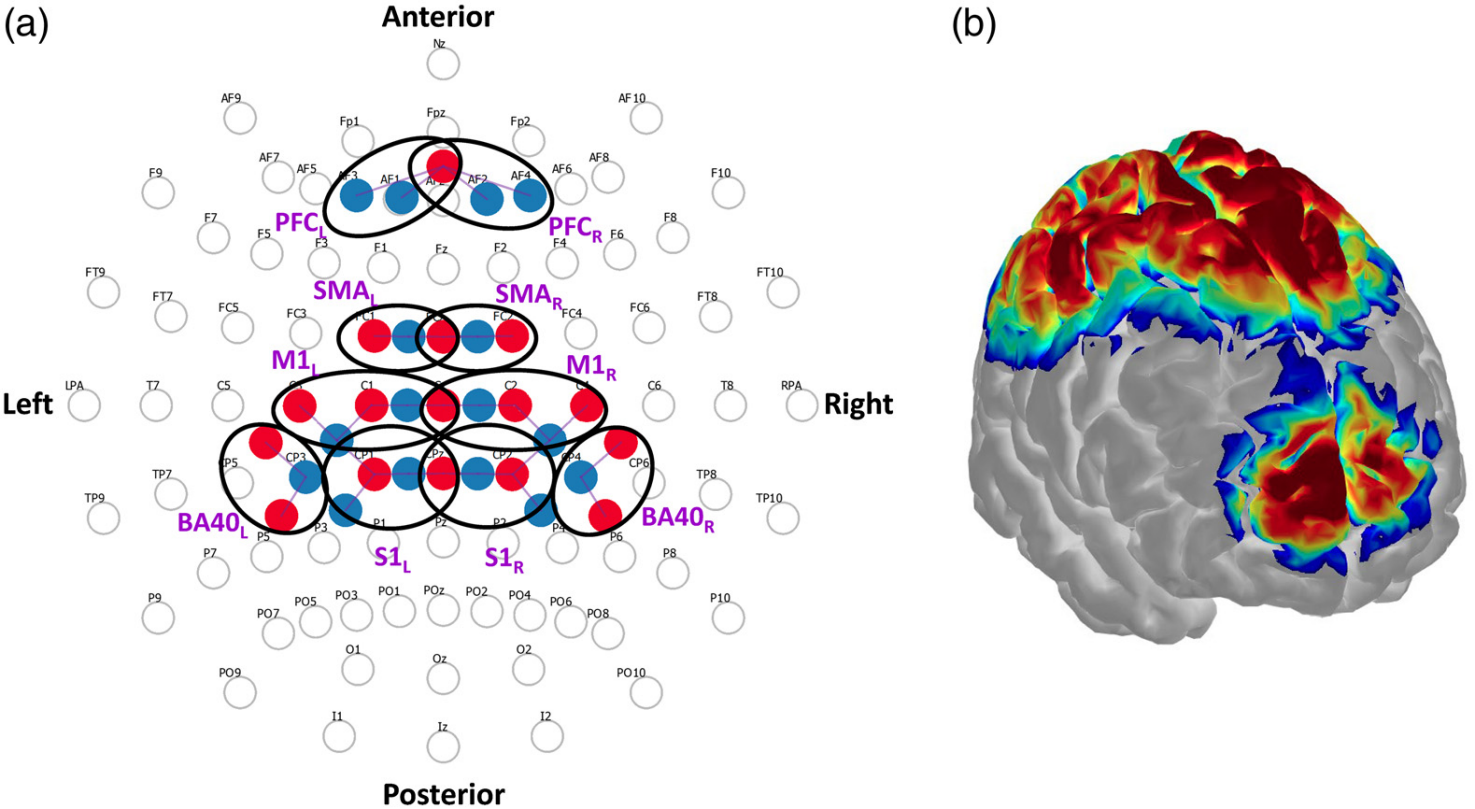
Cortical fNIRS layout and sensitivity map. (a) Geometrical layout of sources (red) and detectors (blue) with respect to the international 10–10 electroencephalogram (EEG) system.^25^ Bold black ovals denote the ROIs, which are subsequently labeled nearby in purple boldface. ROI hemisphere is denoted in subscript. ROIs included PFC, SMA, M1, S1, and BA40. Hemisphere side as well as anterior and posterior of the cranium are noted. (b) Correspondent sensitivity map overlaid onto the Colin27 brain model. Sensitivity computed and displayed with AtlasViewer.^26^

Cortical hemodynamics were measured with a continuous-wave fNIRS instrument (NIRScout, NIRx Technologies, Glen Head, New York) via 16 optical emitters and 16 optical detectors. Each emitter consisted of a dual-wavelength LED (central wavelengths: 760 and 850 nm) directly coupled to the scalp. Each detector was a silicon photodiode collecting backscattered light from the scalp via an optical fiber. The geometrical layout of optical emitters and detectors (collectively referred to as optodes) is shown in Fig. 2(a), alongside the corresponding sensitivity map of the optical probing on the cerebral cortex [Fig. 2(b)] estimated with Monte Carlo-based simulation of photon migration in AtlasViewer.^26^ This configuration resulted in 28 optical channels (i.e., emitter–detector pairings) that interrogated the prefrontal, motor, and somatosensory cortices bilaterally. The geometrical distance between optode pairings ranged from 26 to 37 mm, ensuring the interrogation of the cerebral cortex in all optical channels.^27^

Raw optical signals were collected continuously throughout the experiment at a frequency of 3.91 Hz from all channels at both wavelengths and were subsequently converted to optical density (i.e., logarithm of the raw intensity) and then to concentration changes of HbO and HbR compared to a zeroed baseline according to the modified Beer–Lambert law.^28^^,^^29^ For each channel, HbO and HbR measurements were analyzed separately with a general linear model approach that estimated the scalar weight coefficient (a.k.a., beta weight^30^) of the canonical hemodynamic response that best least-squared fitted the measured hemodynamic response. The general linear model, based on an autoregressive iteratively reweighted least-squares approach, is described in detail in Ref. 31. Of relevance, this method permits correction of motion artifacts and serial correlations within the algorithm [i.e., does not require one to explicitly bandpass filter or apply corrective actions to detect and discard extracerebral, unwanted components, such as systemic oscillations (i.e., cardiac and respiration) and motion artifacts].^30^^,^^32^ For each subject, we considered channels as hemodynamically active if their weight coefficient was statistically different from zero at the significance level of 5%.

At the group level, we used a mixed linear model to estimate the weighting coefficient of all channels in order to determine which of them were hemodynamically active at a statistically significant level. We considered the interaction between the experimental condition (sensory stimulation at high, sensory stimulation at low, motor performance at high force level, and motor performance at low force level) and the group (DM versus control) as the fixed effect contributing to the weight coefficient, while the magnitude of the coefficient of individual subjects was considered as a random effect. Both HbO and HbR were fitted with a positive double-gamma hemodynamic response function (HRF) (canonical HRF), hence, a negative t-value for either HbO or HbR signifies a decrease in respective concentration.

We grouped optical channels into 10 bilateral (right and left) regions of interest (ROIs): prefrontal cortex (PFC), supplementary motor area (SMA), primary motor cortex (M1), primary sensory cortex (S1), and Broadmann Area 40 (BA40), as depicted in Fig. 2(a). We computed individual-level ROI level statistics (weight coefficient, t-value, and p-value).

### 2.7 Statistical Analysis

The data are presented as means ± standard errors (SE). For HbO and HbR, statistically significant individual-level ROI t-scores were compared between “groups” using mixed model analyses of covariance (ANCOVAs) via SPSS 25 (IBM Corporation, Armonk, New York). Between-subject primary factors were group (two levels: DM versus controls). Within-subject factors included “hemisphere” (two levels for the cortex: left and right) and “ROI” (five levels: 1 = PFC, 2 = SMA, 3 = M1, 4 = S1, and 5 = BA40). For monofilament data, main factors included group and nerve (three levels: one level each for the median, radial, and ulnar nerves). For submaximal force production data, main factors included group and level (two levels: 15% and 40% MVC). Evaluation of health state covariates was done to control for health state variability both within and across the two sample groups. ANCOVAs included health state covariates of $A_{1c}$, systolic and diastolic blood pressures, low-density lipoprotein cholesterol, high-density lipoprotein cholesterol, disease duration, menopausal age, body mass index (BMI), PN status (via indicator variable), history of hormone replacement therapy (via indicator variable), and history of treatment with Prempro (via indicator variable). Covariates were selected via automatic linear modeling (ALM) using forward stepwise selection functions in SPSS. Standard SPSS ALM parameters were set at outliers automatically trimmed, single standard model creation, 95% confidence intervals set, use of the AICc information criterion function, and the replication feature enabled. ALM models were run for each measure of interest in order to investigate the influence of health state measures on all behavioral data. In the event of significant covariates determined via ALM and ANCOVA, follow-up correlation analyses were performed between the health state covariate and the measured behavior. Monofilament data were log transformed due to nonlinearity.^33^ Nontransformed values are shown in figures to avoid reader confusion.

## 3 Results

### 3.1 Tactile Evaluation

**Fig. 3:**
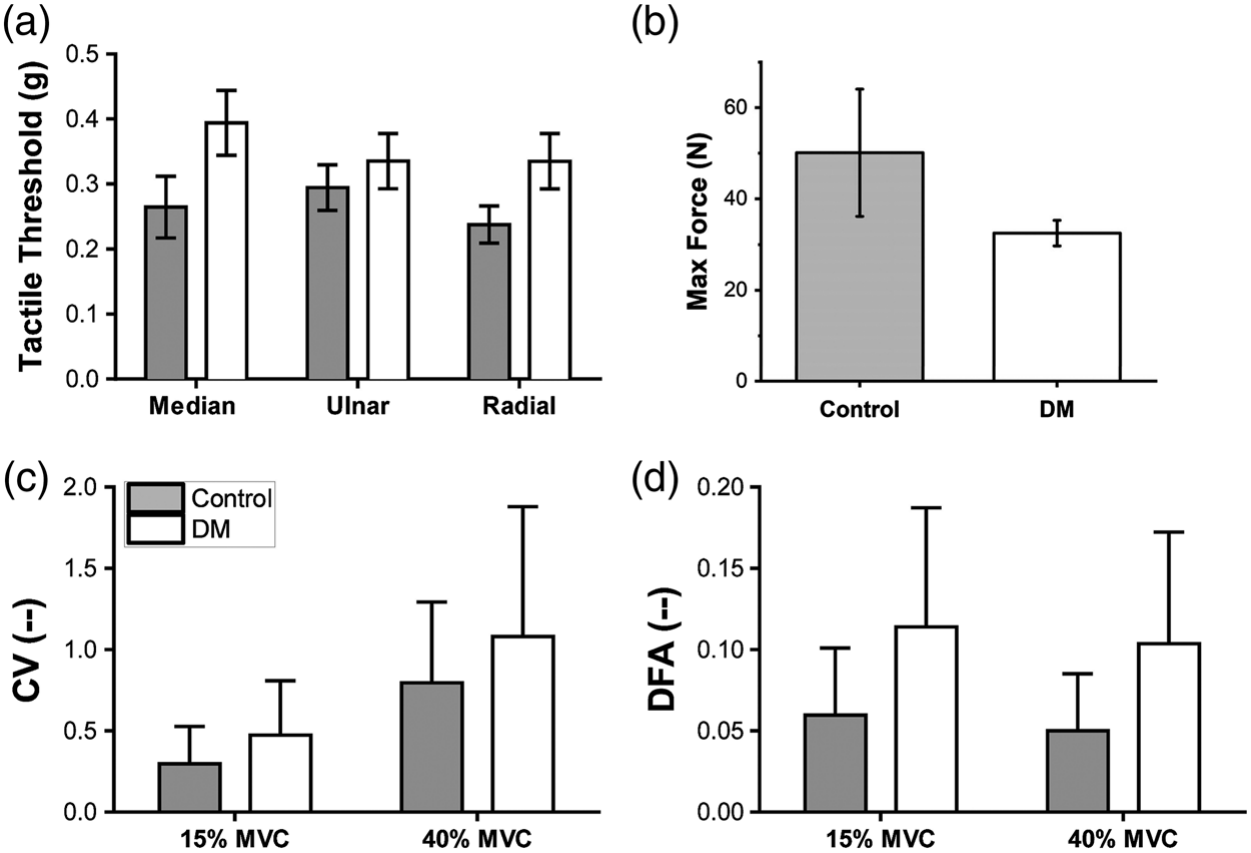
Tactile sensory function data, maximal force data, and indices of submaximal force production. “Group” mean and SE values are shown. (a) Tactile sensory function data for each of the three nerves of the hand, (b) maximal pinch forces, (c) coefficient of variation (CV) in submaximal force production tasks, and (d) DFA in submaximal force production tasks.

A significant difference in tactile detection thresholds was not specifically confirmed between groups (p=0.159); however, the addition of covariates with the statistical model led to a between group difference that approached significance (p=0.098). Tactile detection threshold values did differ among all tested sites [nerve: $F_{2,195}=4.52$, p<0.05, Fig. 3(a)] with a significant difference in the median and radial nerve tactile detection thresholds. Tactile function was generally worse in persons with higher BMI, higher $A_{1c}$, and in those diagnosed with PN, see Table 2 for covariate results.

**Table 2:** Significant ANCOVA covariate output and regression results.

Measure	Covariate	F	p Value	r	p Value
Tactile evaluation	BMI	11.68	<0.001	0.29	<0.001
	$A_{1c}$	4.49	<0.05	0.15	<0.05
	PN status	25.05	<0.001	0.27	<0.001
	Duration	49.56	<0.001	—	—
RMSE	%MVC	199.99	<0.001	—	—
	BMI	7.79	<0.01	—	—
	PN status	6.32	<0.05	—	—
ApEn	HDL cholesterol	4.02	<0.05	—	—
CV	%MVC	2.033	<0.001	—	—
	Total cholesterol	4.29	<0.05	—	—
DFA	BMI	6.95	<0.05	—	—
	Systole	5.77	<0.05	—	—
	Total cholesterol	12.11	<0.001	−0.22	0.058
Digit 1 COP	Menopausal age	5.65	<0.05	—	—

**Note:** %MVC refers to force production requirement level (15% or 40% MVC); $A_{1c}$ refers to glycated hemoglobin; ApEn refers to approximate entropy; BMI refers to body mass index; COP refers to center of pressure; CV refers to coefficient of variation; DFA refers to detrended fluctuation analysis; HDL cholesterol refers to high-density lipoprotein cholesterol; N/A refers to nonapplicable; PN status refers to diabetic peripheral neuropathy diagnosis; and systole refers to systolic blood pressure.

### 3.2 Maximal Force Production

The average force produced during pinch testing was 39.86±6.1N across all participants [Fig. 3(b)]. Neither between group differences nor covariates emerged for maximal force production.

### 3.3 Submaximal Force Production

No group differences were found in RMSE or ApEn measures; instead, health state covariates provided significant effects, see Table 2 for details. Group effects found in CV ($F_{1,61}=4.50$, p<0.05) and DFA measures ($F_{1,61}=12.07$, p<0.001), shown in Figs. 3(c) and 3(d), were replaced by health state covariates when they were added to the statistical model. Covariate results for CV and DFA are also included in Table 2. A finding of differences in contact area of digit 1 (thumb) utilized (via COP measurement^22^^–^^24^) was found with “menopausal age” ($F_{1,51}=5.65$, p<0.05); however, no specific correlation between COP and menopausal age was explicitly found.

### 3.4 Cortical Hemodynamic Responses

#### 3.4.1 Cortical hemodynamic responses during sensory stimulation via vibration

**Fig. 4:**
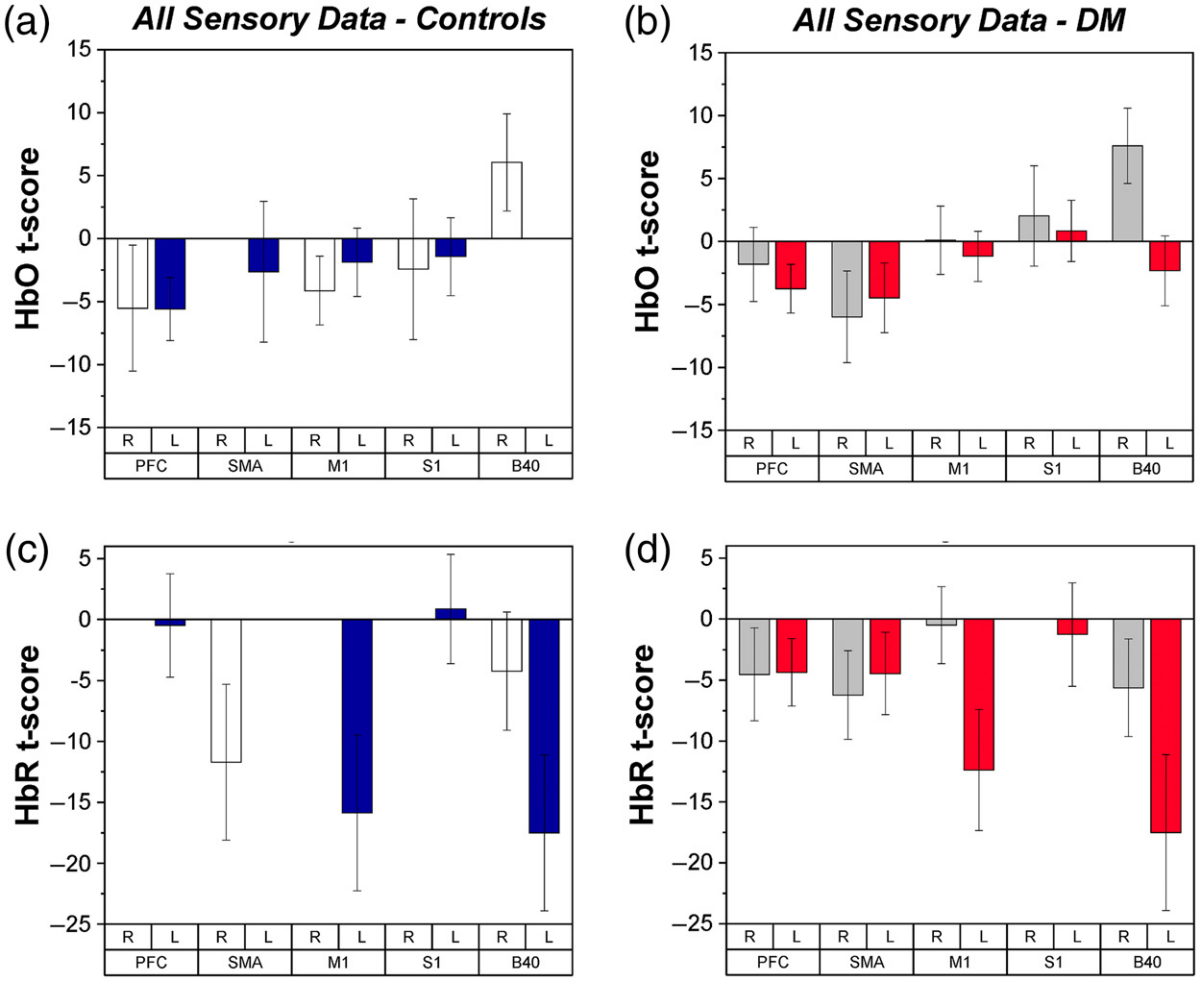
fNIRS t-scores for HbO and HbR during vibrotactile stimulation for each group, as depicted by ROI and hemisphere. Mean and SE values are shown. (a) HbO of controls. White bars indicate right hemisphere and blue bars indicate left hemisphere. (b) HbO of PwDM. Gray bars indicate right hemisphere and red bars indicate left hemisphere. (c) HbR of controls. (d) HbR of PwDM.

Average HbO and HbR data for the sensory stimulation task can be found in Figs. 4(a)–4(d). HbO did not show significant effects with respect to group (p>0.5), hemisphere (p>0.5), or region (p>0.2)*.* HbR did not show significant effects with respect to group (p>0.8) or region (p>0.2); however, a significant effect of hemisphere ($F_{1,11}=6.03$, p<0.05) was found such that a larger increase in HbR was found for the left hemisphere as compared to the right hemisphere, consistent with stimulation of the right hand [Figs. 4(c) and 4(d)]. HbO and HbR values were found to correlate with some measures of health state, as reported in Table 3.

**Table 3:** Significant correlation results for HbO and HbR measures.

Measure	Correlate	r	p Value
HbO (sensory stimulation)	Duration	−0.409	<0.005
	Prempro history	−0.325	<0.05
HbR (sensory stimulation)	PN status	−0.333	<0.05
	Prediabetes history	−0.313	0.055
HbO (motor tasks)	$A_{1c}$	−0.44	<0.001
	ApEn	0.185	<0.05
	BMI	−0.350	<0.001
	PN status	−0.252	<0.005
	Total cholesterol	0.310	<0.001
HbR (motor tasks)	PN status	−0.21	<0.05

**Note:** $A_{1c}$ refers to glycated hemoglobin; ApEn refers to approximate entropy; BMI refers to body mass index; HbO refers to oxygentated hemoglobin; HbR refers to deoxygentated hemoglobin; and PN status refers to diabetic peripheral neuropathy diagnosis.

#### 3.4.2 Cortical hemodynamic responses during manual motor performance

**Fig. 5:**
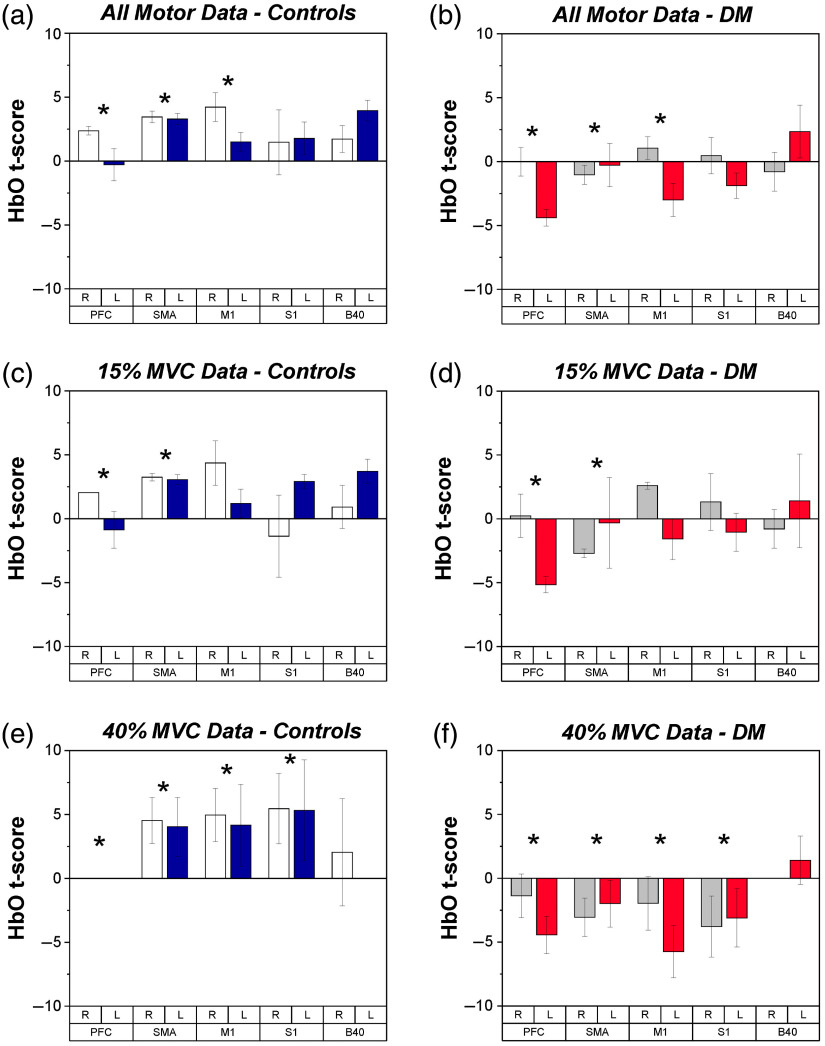
fNIRS t-scores for HbO during submaximal force production tasks for each group, depicted by ROI and hemisphere. Mean and SE values are shown. * indicates a significant difference between groups. (a) HbO of controls across all motor tasks. White bars indicate right hemisphere and blue bars indicate left hemisphere. (b) HbO of PwDM across all motor tasks. Gray bars indicate right hemisphere and red bars indicate left hemisphere. (c) HbO of controls during 15% MVC force production tasks. (d) HbO of PwDM during 15% MVC force production tasks. (e) HbO of controls during 40% MVC force production tasks. (f) HbO of PwDM during 40% MVC force production tasks.

During the manual motor tasks, significant effects of group ($F_{1,106}=9.04$, p<0.005) and a Hemisphere × Region interaction ($F_{4,106}=3.51$, p<0.05) were found in HbO, as shown in Figs. 5(a) and 5(b). Overall, the data indicate significantly larger HbO values in controls versus PwDM, particularly in the left hemisphere during motor tasks. Notably, PFC, SMA, M1, and S1 were generally lower in terms of HbO in PwDM via Bonferroni corrected *post-hoc*. HbO values were found to correlate with some measures of health state and motor performance, as reported in Table 3.

**Fig. 6:**
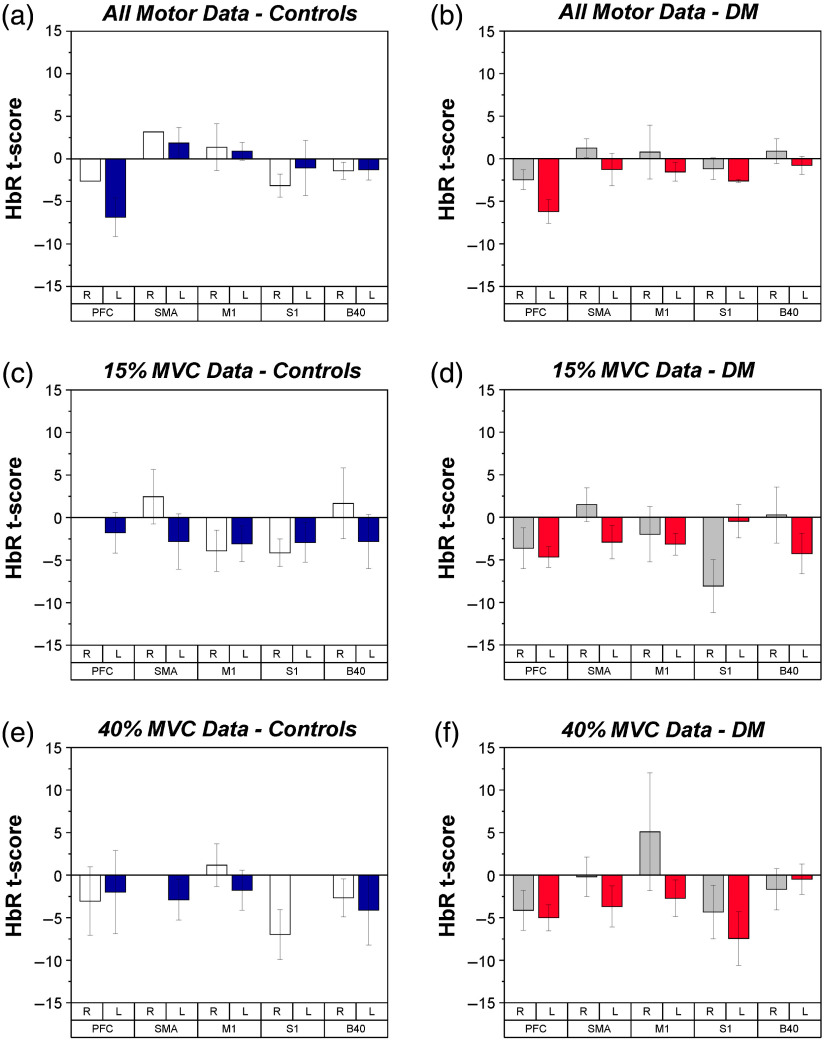
fNIRS t-scores for HbR during submaximal force production tasks for each group, depicted by ROI and hemisphere. Mean and SE values are shown. (a) HbR of controls across all motor tasks. White bars indicate right hemisphere and blue bars indicate left hemisphere. (b) HbR of PwDM across all motor tasks. Gray bars indicate right hemisphere and red bars indicate left hemisphere. (c) HbR of controls during 15% MVC force production tasks. (d) HbR of PwDM during 15% MVC force production tasks. (e) HbR of controls during 40% MVC force production tasks. (f) HbR of PwDM during 40% MVC force production tasks.

Additional analyses of HbO indicated a significant difference in cortical HbO use between controls and PwDM, particularly in the low force production task (15% MVC). Significant effects of group ($F_{1,39}=6.11$, p<0.05) and a Hemisphere × Region interaction ($F_{4,39}=2.81$, p<0.05) were found in HbO during the 15% MVC task, as shown in Figs. 5(c) and 5(d). These effects are similar to that for all HbO data for the manual motor tasks. Notably, no major group, region, hemisphere effects, or interactions were found in the initial analysis of the 40% MVC task; however, an impact of menopausal age ($F_{1,32}=6.09$, p<0.05) on HbO was found which helped reveal a group ($F_{1,32}=12.76$, p<0.005) effect within the 40% MVC task, as shown in Figs. 5(e) and 5(f). While the correlation between menopausal age and HbO data was not significant, the models indicate a negative relationship between the two measures.

During the manual motor tasks, a significant effect of region ($F_{4,74}=2.66$, p<0.05) was found in HbR, as shown in Fig. 6. *Post-hoc* analysis indicates that the PFC was overall more active than SMA, S1, and B40 during motor tasks. HbR values were found to correlate with some measures of health state, as reported in Table 3. Separate analyses of HbR by %MVC did not yield any significant results.

## 4 Discussion

The purpose of the current study was to evaluate changes in cortical oxygenation indices of postmenopausal women both with and without DM during (1) tactile stimulation of the hands and (2) manual force production tasks. In terms of our hypotheses, the data support (at least in part) each of our two primary hypotheses. With respect to Hypothesis #1, HbO values differed between groups during force production tasks while HbR did not show group effects in either vibrotactile stimulation or force production tasks. In support of Hypothesis #2, decline in motor function output was observed in both tasks in PwDM as compared to controls; however, tactile detection thresholds did not differ between groups. With respect to our exploratory arm of the study, there is substantial evidence of the influence of poor health state as an impact on all measures of interest in this study. In the following paragraphs, we discuss the results of this study in regard to the published literature as it relates to cortical oxygenation and functional neuroimaging, possible sex-based differences in PwDM, and the impact of health state markers in assessment of both behavior and cortical function.

### 4.1 Functional Cortical Activity and Tissue Oxygenation

The primary outcome of this manuscript indicates a significant global difference in the use of HbO between postmenopausal female PwDM and age- and sex-matched controls. Specifically, cortical use of HbO was significantly higher in controls during the performance of motor tasks as compared to PwDM across most ROIs, suggesting that all aspects of sensorimotor function (including planning and executive function) may be directly impacted by DM. This finding coupled with simultaneous deficits in fine motor function in PwDM indicates a potential cortical root for motor performance degradation in PwDM. This is the first evidence of a cortical contribution for motor dysfunction in PwDM, whereas the traditional point of view is that peripheral nerve damage solely accounts for any motor deficits in this population.^34^^–^^36^ Interestingly, HbO differences in the fine motor function task (15% MVC force production) suggest that tasks involving finer manual function control may be more negatively impacted by DM at the cortical level. This may have significant influence over the performance of activities of daily living (ADLs), as many ADLs are manual tasks involving low levels of force production and control.

Our finding of reduced cortical use of HbO is certainly notable, as HbO use is not indicated by other functional imaging techniques—most specifically functional magnetic resonance imaging (fMRI)—as they are not sensitive to HbO. By its nature, HbO is diamagnetic (containing no unpaired electrons) and is thus not attracted to any magnetic field. Techniques, such as fMRI, rely on the paramagnetism of HbR, thereby not fully representing potential issues with cortical hemodynamic responses, which involve both HbO and HbR.

As cortical HbR has been shown to increase in proportion to manually produced forces,^37^^,^^38^ it is unlikely that the null HbR results in the motor tasks are due to the level of submaximal forces required in the current study. We acknowledge that HbR use has been found to increase during fatiguing tasks;^39^ currently, it is unknown if this phenomenon is consistent for PwDM. This provides an opportunity for immediate future investigation.

Reduced use of HbO in the cortex during motor tasks in PwDM may indicate reduced bioavailability of oxygen within the cortex. This observation is associated with the occurrence of motor impairment in the current data set. Currently, it is unclear as to why exactly this occurs. The evidence base indicates that DM is associated with increased hemoglobin-oxygen affinity, which is responsible for reduced oxygen delivery to tissues within the body.^40^ However, DM is also associated with impaired hyperemic responses, endothelial dysfunction, and microvascular dysfunction.^13^^,^^16^^,^^41^^,^^42^ DM has also recently been linked to pathological neurovascular decoupling in PwDM (as reviewed in Ref. 43) and conflicting reports of reduced cerebral blood flow.^44^^,^^45^ There is the possibility that impaired active hyperemia and changes in overall blood oxygenation both contribute to the deficits we have observed in PwDM. However, this cannot be resolved by the current data set, but the data do suggest that further mechanistic investigation is warranted into the neurovascular and potential reduced blood flow roots of reduced cortical HbO in female PwDM.

### 4.2 Influence of Sex on Cortical Structure/Function

Contrary to our previous findings,^5^^,^^12^ differences in tactile function and maximal force profiles between the current sample of postmenopausal PwDM and age- and sex-matched controls were not found. On the surface, this result is surprising; however, this particular study focused on postmenopausal women, whereas our previous work included a cross-section of participant ages, including both males and females. It is possible that sex-based differences in sensorimotor function exist in PwDM, particularly as age increases, as supported by Ref. 33. Sex-based difference in the presentation of DM complications is an emerging area of interest in metabolic research.^46^ Our recent work indicates a significant impact of sex, age, handedness, and metabolic health on the development of poor tactile sensation.^33^ While the presence of DM and PN does adversely impact overall tactile function, sex-based differences continue to persist^33^ such that females present with less impairment in behavioral measures as compared to males, reinforcing the behavioral results within the current data set. The root of these sex-based differences may stem from several sources, including differences in sex hormone levels (e.g., testosterone, estrogen, and other androgens), sexual dimorphism and asymmetry of the cortex,^47^^–^^49^ and reports of persistent metabolic youth in the aging female brain as compared to males.^50^ Reduced cortical asymmetry in females may also partially explain the absence of strong hemispheric differences in hemodynamic responses within the current data set.^47^

Decreases in hyperemic responses have also been found in postmenopausal females as compared to males (both without DM), indicating a baseline difference in blood flow due to sex, which may impact both sensory and motor functions.^51^^–^^53^ There is some evidence that estrogen-based hormone replacement therapy (HRT) improves hyperemic responses in postmenopausal females;^54^^,^^55^ but there is no indication that it is protective against deficits induced by DM. Discontinued use of conjugated estrogen HRT in the early 2000s due to cardiovascular complications of the treatment (e.g., Prempro) further complicates the picture.^56^ Specifically, Prempro use introduces significant additional cardiovascular disease risk in females. In the current data set, reduced cortical oxygenation along with motor deficits was demonstrated in PwDM as compared to controls. These results were not impacted by HRT status (both HRT history overall and specifically by Prempro use), indicating cortical deficits linked to DM without a directional influence of HRT in postmenopausal females.

### 4.3 Influence of Health State on Behavioral and Cortical Activation Measures

Additionally, significant influences of overall poor health state were found across the data set. Specifically, measures such as high $A_{1c}$, high blood pressure, high BMI, and high cholesterol, appeared as detrimental factors in the current data set. This suggests that overall cardiovascular health may be a significant factor in the interplay between cortical activity and overt sensorimotor behaviors. We acknowledge that a history of PN did appear as an influence in some of the statistical models; however, it did not account for the between-group differences found; instead, it enhanced the effects. This suggests that nonperipheral factors do indeed relate to sensorimotor dysfunction in PwDM contrary to prior clinical points of view.^34^^–^^36^ Overall, our data indicate a negative impact of poor health state, independent of specific DM medical management, that is a significant contributor to reduced functional use of HbO by the cortex.

### 4.4 Limitations

We acknowledge that the present study does not account for systemic changes that may have occurred within the data set [short-term physiological changes (e.g., respiration)]. Despite our instructions to breathe normally, it is possible that during the motor tasks, participants held their breath while exerting the force, which may have impacted the fNIRS signals. In future work, we recommend adding additional measures of blood oxygenation and respiration rates in order to evaluate this possibility.

### 4.5 Conclusion

Overall, the data demonstrate reduced use of cortical HbO in PwDM while they simultaneously showed deficits in manual motor tasks, providing the first evidence of functional cortical activity deficits relating to motor dysfunction in this population. Functional cortical activation deficits were not specifically noted in HbR. Similar effects were not found in evaluation of sensory function. Health state indices were found to clarify group effects. Further work is needed to clarify potential sex-based differences in PwDM and the root of reduced cortical oxygenation use in PwDM during motor tasks.

## Code, Data, and Materials Availability

Data analyzed in this project will be available via Zenodo (https://zenodo.org/) upon manuscript publication. As public sharing of protected health information, such as date of birth, date of diagnosis, dates of treatment, and other private medical history information pertinent to each participant, may violate HIPAA and the Texas Medical Privacy Act, these data will not be shared to protect patient identity.
